# Sex-Differential Effect on Infant Mortality of Oral Polio Vaccine Administered with BCG at Birth in Guinea-Bissau. A Natural Experiment

**DOI:** 10.1371/journal.pone.0004056

**Published:** 2008-12-29

**Authors:** Christine Stabell Benn, Ane Bærent Fisker, Amabelia Rodrigues, Henrik Ravn, Erliyani Sartono, Hilton Whittle, Maria Yazdanbakhsh, Peter Aaby

**Affiliations:** 1 Bandim Health Project, Statens Serum Institut, Copenhagen, Denmark; 2 Bandim Health Project, Indepth Network, Bissau, Guinea-Bissau; 3 Department of Immunoparasitology, Leiden University Medical Centre, Leiden, The Netherlands; 4 The MRC Laboratories, Fajara, The Gambia; CIET, Canada

## Abstract

**Background:**

The policy to provide oral polio vaccine (OPV) at birth was introduced in low-income countries to increase coverage. The effect of OPV at birth on overall child mortality was never studied. During a trial of vitamin A supplementation (VAS) at birth in Guinea-Bissau, OPV was not available during several periods. We took advantage of this “natural experiment” to test the effect on mortality of receiving OPV at birth.

**Methodology:**

Between 2002 and 2004, the VAS trial randomised normal-birth-weight infants to 50,000 IU VAS or placebo administered with BCG. Provision of OPV at birth was not part of the trial, but we noted whether the infants received OPV or not. OPV was missing during several periods in 2004. We used Cox proportional hazards models to compute mortality rate ratios (MRR) of children who had received or not received OPV at birth.

**Principal Findings:**

A total of 962 (22.1%) of the 4345 enrolled children did not receive OPV at birth; 179 children died within the first year of life. Missing OPV at birth was associated with a tendency for decreased mortality (adjusted MRR = 0.69 (95% CI = 0.46–1.03)), the effect being similar among recipients of VAS and placebo. There was a highly significant interaction between OPV at birth and sex (p = 0.006). Not receiving OPV at birth was associated with a weak tendency for increased mortality in girls (1.14 (0.70–1.89)) but significantly decreased mortality in boys (0.35 (0.18–0.71)).

**Conclusions:**

In our study OPV at birth had a sex-differential effect on mortality. Poliovirus is almost eradicated and OPV at birth contributes little to herd immunity. A randomised study of the effect of OPV at birth on overall mortality in both sexes is warranted.

## Introduction

In low-income countries, the World Health Organization (WHO) recommends oral polio vaccine (OPV) at birth and in 3 doses together with diphtheria-tetanus-pertussis (DTP) vaccines at 6, 10, and 14 weeks of age [1]. Furthermore, OPV has been delivered in campaigns to children aged 6 months to 5 years of age as part of the attempt to eradicate polio infection. The policy to provide OPV at birth was introduced more than 20 years ago to increase the coverage for OPV [2]. OPV at birth might also be associated with increased antibody titres [3], [4]. However, the effect of OPV at birth on overall child mortality was never studied.

Routine vaccinations in childhood may have non-specific and sex-differential effects on overall mortality [5]–[8]. The effects are large and may strongly influence overall mortality and seriously distort female- male mortality ratios in high-mortality settings. Our previous work has concentrated on measles vaccine (MV) and BCG vaccine, which are associated with decreased overall mortality and decreased female-male mortality ratios [5], [6], and DTP vaccine, which has been associated with increased female-male mortality ratios [7], [8]. Furthermore, we also conducted one study of an OPV campaign; receiving OPV in the campaign compared with not receiving OPV was associated with significantly decreased mortality among the youngest children [9]. At the national hospital in Guinea-Bissau, following periods with DTP being unavailable, patients having received OPV only and no DTP had significantly lower in-hospital mortality than patients having received both the recommended DTP and OPV [10]. The results suggest that OPV might also have non-specific beneficial effects on mortality.

We recently conducted a large vitamin A supplementation trial in Guinea-Bissau. Children were randomised to 50,000 IU vitamin A or placebo when they received BCG after delivery [11]. Hence, all children received BCG at enrolment. All children should also receive OPV according to current recommendations. However, during the last year of the trial the national immunization programme experienced several periods during which OPV was not available. We took advantage of this “natural experiment” to test the effect of receiving OPV at birth on infant mortality. Our *a priori* hypothesis was that not receiving OPV at birth would increase mortality, particularly for girls.

## Methods

### Setting

The Bandim Health Project (BHP) has a demographic surveillance system (DSS) which covers approximately 90,000 inhabitants in six suburban districts of the capital of Guinea-Bissau. All houses are visited monthly to register new pregnancies and births. Children are followed with home visits every third month to register vaccinations, infections, hospitalisations, feeding patterns and survival. The study area is economically poor. Most people live in multi-family mud-brick houses with zinc or straw roofs. More than 60% have no electric installations in the house. Around 30% of the mothers in the present trial had no education. During the trial, the prevalence of HIV-1 was around 5% in the study area among women of fertile age [12]. A large proportion of pregnant women are screened for HIV to prevent vertical transmission. With the vertical-transmission control program ongoing, we expect less than 1% of the children to be HIV-1 infected.

### Vitamin A trial

#### Participants

As described in detail elsewhere [11] the vitamin A trial took place from November 13, 2002 to November 28, 2004. It enrolled infants who came for the BCG vaccination recommended at birth. The inclusion criteria were weight ≥2500 g at presentation for BCG vaccination and no signs of overt illness/malformations. It was not a criterion that the child should receive OPV at birth. Mothers giving birth at the maternity wards at the national hospital and the local health centre were offered BCG and enrolment immediately after the delivery. Mothers who delivered at home were invited to participate when they came for BCG at two of the three health centres in the study area. The third health centre in the study area was not enrolling children for logistic reasons. A total of 1181 (27%) of the children came after the first week of life; their median age at enrolment was 16 days (10–90 centiles = 9–64 days).

#### Randomisation

Details regarding the trial were explained to the mother by a trained assistant, who also asked the mother if she had any questions about the study. Oral consent was obtained. Written consent was obtained if the mother knew how to write her own name. If not, a statement that she had understood the information given and agreed to participate was signed by the assistant. The mother drew a lot from an envelope prepared by the study supervisor. Each envelope contained 100 lots, 50 marked “1”, and 50 marked “2”, indicating from which of two numbered bottles, “1” or “2”, the child should receive its supplement. Hence, half of the children received 50,000 IU vitamin A as retinyl palmitate and 20 IU vitamin E in ½ ml vegetable oil, the other half received only 20 IU vitamin E in ½ ml vegetable oil. The code was only opened when all children had reached 12 months of age.

#### Outcomes examined

All children were followed through the DSS every third month and were visited by a special team at 12 months of age. Deaths were registered at each visit and followed by a verbal autopsy conducted by a trained local physician. The physician used a questionnaire designed by the INDEPTH network [13]. Based on the questionnaire a panel of three medical doctors, including at least one local doctor, all being ignorant of the randomisation group, reached consensus on the cause of death. If consensus could not be reached a fourth doctor was consulted. Deaths were categorised based on suggestions from the WHO [14]. No death within the first year was due to accident. The BHP registers hospitalisations of children from the study area at the paediatric department of the nearby national hospital. By linking the trial database with the hospitalisation database we were able to identify trial children who had been hospitalised.

### Current study

**Figure 1:**
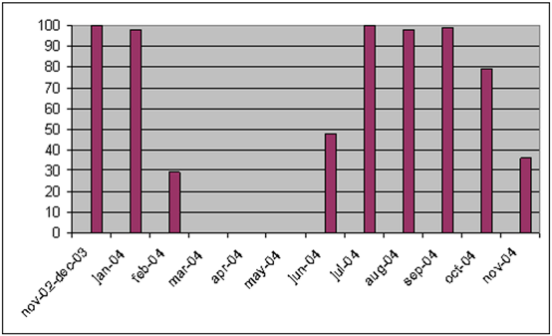
Percentage of enrolled children who received OPV at birth during a vitamin A trial from 2002–2004 in Guinea-Bissau.

All trial infants were vaccinated intradermally in the upper left deltoid region with 0.05 ml BCG vaccine (Statens Serum Institut, Copenhagen, Denmark). The BHP provided BCG vaccine for the trial participants. According to recommendations all infants should also receive OPV at birth. OPV is administered orally. During the study three types of OPV were used in Guinea-Bissau, produced by Novartis, Sanofi Pasteur, and GlaxoSmithKline. All vaccines were supplied through the national immunization programme. However, OPV was missing during periods of the trial. Hence, the children who were BCG vaccinated during these periods would only receive BCG and not OPV. As part of our trial routines, we noted at the inclusion form whether the children received OPV or not. Based on the distribution of children who had not received OPV, OPV was missing at one or more inclusion sites 1) from the beginning of February 2004 to the beginning of June 2004, 2) briefly in late June 2004, and 3) from mid-October 2004 to the trial enrolment ended in November 2004 (Figure 1). Hence, all non-OPV recipients were enrolled in 2004. We tested the mortality levels among OPV recipients in 2002, 2003, and 2004. The mortality levels were the same during all three years (p = 0.754). Furthermore, the female-male mortality rate ratio among OPV recipients was the same throughout the whole study period (p = 0.799). Hence, we included all children in the analysis.

### Statistical analysis

We compared baseline characteristics of children who had or who had not received OPV at birth in a logistic regression analysis including all variables in order to identify important potential confounders, which could be adjusted for in subsequent analyses.

In the main analyses we studied the effect of *not* receiving OPV at birth on the following three outcomes: mortality, hospitalisation and risk of dying when hospitalised (hospital case-fatality). All analyses were conducted including all children as well as stratified by sex, and without and with adjustment for potential confounders.

#### Mortality

We compared mortality rates of children who had or who had not received OPV at birth in Cox proportional hazards models [15] providing mortality rate ratios (MRR) with 95% confidence intervals (CI). Age was used as the underlying time. Hence, age is inherently controlled for in all analyses. Infants provided follow-up time from enrolment until they reached 12 months of age, moved, or died.

#### Hospitalisation

Incidence rates of hospitalisation were also compared in Cox models with age as the underlying time providing incidence rate ratios (IRR) with 95% CI. Multiple events were allowed, accounting for dependence within individuals using robust variance estimates. Infants provided follow-up time from enrolment until they reached 12 months of age, moved, or died.

**Hospital case-fatality** was compared using a Poisson regression model with robust variance estimates providing relative risks with 95% CI [16].

We aimed to study potential effect modification. Effect modification was analysed by investigating the homogeneity of the effect of OPV in the different categories of the suspected modifier using Wald test statistics. Hence, we obtained p-values for equal effect of receiving or not receiving OPV in different strata of a covariable. When addressing the effect of OPV according to birth weight and arm circumference, we restricted the analysis to children enrolled on day 0 and 1 (N = 2572) for whom information on birth characteristics was available and dichotomised the anthropometric measurements into the lowest quartile and the three highest quartiles.

Test for proportionality of hazard rates were computed using Schoenfeldt residuals and by visual inspection of the Nelson-Aalens estimator.

## Results

A total of 962 children (22.1%) of the 4345 enrolled children did *not* receive OPV at birth (Appendix S1). There were significant differences between those who did and those who did *not* receive OPV at birth (Table 1). *Not* having received OPV at birth was associated with low age at enrolment, no maternal school education, low arm circumference, being enrolled at the Belem Health Centre, and dry season (Table 1). Missing OPV at birth was not associated with length, maternal arm circumference or the frequency of subsequent vaccinations (data not shown).

**Table 1:** The association between background factors and receiving OPV at birth.

	No OPV at birth (N = 962)	OPV at birth (N = 3383)	P
	N (%)	N (%)	
Male sex	503 (52%)	1697 (50%)	P = 0.245
Rainy season	262 (27%)	1892 (56%)	P<0.0001
Suburb*			P = 0.048
Bandim	454 (47%)	1735 (51%)	
Other	495 (51%)	1591 (47%)	
Maternal school education*			P<0.0001
Yes	586 (61%)	2065 (61%)	
No	332 (35%)	930 (27%)	
Maternal ethnicity*			P = 0.199
Pepel	254 (26%)	957 (28%)	
Other	683 (71%)	2311 (68%)	
Electricity*			P = 0.376
Yes	311 (32%)	1101 (33%)	
No	627 (65%)	2168 (64%)	
Place of enrolment			P = 0.065
Hospital	533 (55%)	1953 (58%)	
Bandim HC	316 (33%)	1117 (33%)	
Belem HC	113 (12%)	313 (9%)	
Age at enrolment			P = 0.314
<1 week	576 (60%)	1996 (59%)	
1–5 weeks	345 (36%)	1201 (36%)	
6 weeks+	41 (4%)	186 (6%)	
Weight lowest quartile	159 (28%)	486 (24%)	P = 0.104
Arm circumferences lowest quartile	228 (24%)	565 (17%)	P<0.0001
Randomised to vitamin A	482 (50%)	1663 (49%)	P = 0.604

* Figures do not add up due to lacking information.

### Mortality

A total of 179 children died within the first year of life. Missing OPV at birth was associated with a tendency for decreased mortality from enrolment to age 12 months (Table 2). There was a highly significant interaction between OPV at birth and sex; missing OPV was associated with a weak tendency for increased mortality in girls, but significantly decreased mortality in boys (Table 2). Adjustment for the background factors which were associated with *not* having received OPV had essentially no impact on the overall and sex-specific estimates or the interaction term (Table 2). In other words, the association between OPV at birth and sex meant that girls had 153% higher mortality than males in the group that did *not* receive OPV at birth (female-male MRR = 2.53 (1.16–5.55)) but 20% lower mortality among children who had received OPV at birth (0.80 (0.58–1.11)).

**Table 2:** The effect of missing OPV at birth on mortality up to 12 months of age.

	No OPV at birth	OPV at birth	No OPV vs. OPV MRR (95% CI) (crude)	No OPV vs. OPV MRR (95% CI) (adjusted)
	MR (Deaths/pyrs)	MR (Deaths/pyrs)		
**All**	34.9 (30/859)	49.2 (149/3031)	0.71 (0.48–1.05)	0.69 (0.46–1.03)
**Boys**	20.0 (9/451)	54.1 (82/1514)	**0.37 (0.18–0.73)**	**0.35 (0.18–0.71)**
**Girls**	51.5 (21/408)	44.2 (67/1517)	1.16 (0.71–1.90)	1.14 (0.70–1.89)
**P for interaction**			**0.008**	**0.006**

* adjusted for season, suburb, maternal education, place of enrolment, lowest quartile arm circumference.

Note: MR = mortality rate, MRR = mortality rate ratio, pyrs = person-years of risk.

There were two major periods without OPV. Among children enrolled from January 2004–June 2004, the overall adjusted estimate was 0.72 (0.38–1.38); 0.52 (0.18–1.49) in boys and 0.87 (0.38–2.00) in girls. Among children enrolled from July 2004 and until the end of the trial in November 2004 it was 0.62 (0.22–1.80); 0.24 (0.03–1.86) in boys and 1.22 (0.34–4.44) in girls.

**Figure 2:**
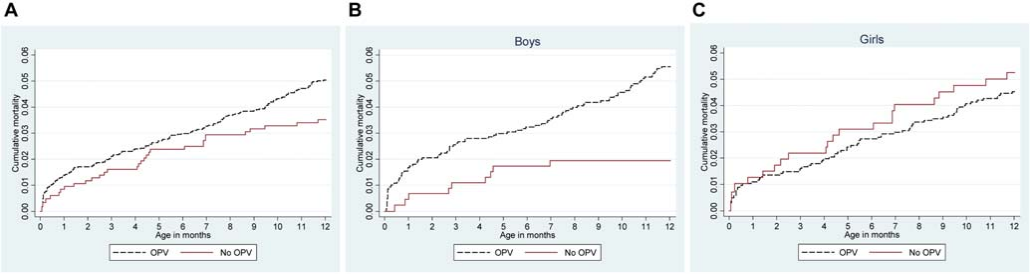
A–C. Cumulative mortality curves as a function of receiving OPV at birth or not, overall (A) and by sex (B,C).

The cumulative mortality curves for all children and for boys and girls separately, are illustrated in Figures 2A–C. The effect of not receiving OPV at birth on mortality up to 2 months of age (53 deaths) was 0.39 (0.12–1.29) among boys and 1.38 (0.54–3.57) among girls (p = 0.099). However, the effect of not receiving OPV at birth was not limited to the first months of life, but appeared to continue throughout the first year of life.

The effect of missing OPV at birth was the same in the vitamin A and placebo groups (data not shown). The effect did not vary with age at inclusion (data not shown). Furthermore, the effect did not vary with place of enrolment (health centres versus hospital), or by season (data not shown). The negative effect of missing OPV was seen in boys of educated as well as mothers without any schooling (data not shown), and in children with lower birth weight and arm circumference as well as those without (data not shown).

We studied the effect of missing OPV at birth on the major causes of death: malaria, diarrhoea, respiratory diseases and septicaemia; 58 deaths had other less common causes of death or had no verbal autopsy conducted because the family had moved. Though none of the differences reached statistical significance, the sex-differential effect of OPV at birth was seen for all four major causes of death (Table 3).

**Table 3:** The effect of missing OPV at birth on disease-specific mortality up to 12 months of age.

	All	No OPV versus OPV Adjusted MRR (95% CI)	
		Boys	Girls
**Malaria** (35 deaths)	0.70 (0.28–1.73)	0.27 (0.03–2.06)	1.05 (0.38–2.91)
**Diarrhoea** (34 deaths)	1.21 (0.53–2.79)	0.53 (0.12–2.37)	2.12 (0.77–5.85)
**Respiratory infection** (26 deaths)	0.64 (0.21–1.89)	N/A (0/503 vs. 10/1697)	1.23 (0.38–3.91)
**Septicaemia** (26 deaths)	1.15 (0.45–2.94)	0.99 (0.27–3.61)	1.35 (0.36–5.11)

* adjusted for season, suburb, maternal education, place of enrolment, lowest quartile arm circumference.

Note: MRR = mortality rate ratio.

### Hospitalisations

Among study children, a total of 366 hospitalisations took place from enrolment to 12 months of age. We found no effect of missing OPV at birth on the risk of hospitalisations within the first year in either sex; the adjusted IRR among boys being 0.84 (0.59–1.20) and 1.05 (0.68–1.63) among girls. The effect of missing OPV at birth on hospital case-fatality also did not differ between boys and girls, the adjusted relative risks being 1.04 (0.45–2.40) and 1.30 (0.55–3.07), respectively.

## Discussion

The WHO recommends OPV at birth. The effect of OPV on mortality has never been investigated. In our study, missing OPV at birth was associated with significantly reduced mortality among boys but a weak tendency for increased mortality among girls, indicating that OPV at birth may have a sex-differential effect on mortality.

### Bias and confounding factors

The study was observational. Though it was a “natural experiment” in the sense that it was logistic that determined the availability of OPV there was nonetheless more children of uneducated mothers and with low arm circumference who did *not* receive OPV. These children may have had a higher risk of dying during the first year of life *a priori*. However, control for maternal education and arm circumference did not change the estimates much. Also, if it was the less well-off children who did *not* receive OPV, it would not explain the significantly lower mortality among boys who did *not* receive OPV. In conclusion, we do not think that there is any plausible uncontrolled background factor, which may explain our observation.

### Consistency of the findings

It strengthens the observation that the effect of *not* receiving OPV was comparable during the two major independent periods without OPV. Though no significant associations were observed between *not* receiving OPV at birth and major causes of death, hospitalization or hospital case-fatality, the pattern was the same. For all major causes of death there was lower mortality for boys and higher for girls if they had *not* received OPV; for both hospitalization and case-fatality there was a 20–25% difference between boys and girls which went in the same direction as the overall result. Hence, the results are compatible with the hypothesis that OPV at birth increases mortality of infant boys. Many factors influence a mother's choice to bring the child to the hospital, and hospitalizations may not be the best proxy for severe disease. The vast majority of children died outside the hospital.

### Sex-differential effects

We have found sex-differential effects for almost all routine vaccinations. Our previous studies of OPV did not show any strong sex-differential effects [9], [10]. In the present study the sex-differential effect was pronounced and worrying because it suggested a negative effect of OPV at birth in boys. We have no biological explanation for the sex-differential effect. However, the consistent observation of sex-differential effects on overall mortality of routine vaccinations such as DTP and measles vaccine does add plausibility to the hypothesis that boys and girls may have different immune systems, which are affected in a differential manner by our current interventions.

### Conclusion

OPV given with BCG at birth became policy for logistic reasons. Subsequently a single study showed that it may increase antibody titres. The effect of providing OPV at birth on overall mortality was never tested. Many routine vaccines have non-specific effects on mortality that affects boys and girls differently. This “natural experiment” suggests that the same may apply to OPV administered with BCG at birth. Not receiving OPV at birth was associated with a tendency for increased mortality in girls, but significantly decreased mortality in boys. There is political interest in substituting OPV with IPV [17]. However, this may take many years. If OPV at birth has unwarranted effects in boys, provision of OPV at birth to boys should be stopped since OPV at birth probably contributes little to immunity. If OPV has beneficial effects in girls, substituting with IPV may increase overall mortality in girls. Randomised studies of the effect of providing OPV at birth on overall mortality in both sexes are warranted.

### Ethical approval

The protocol was presented to the Ministry of Health in Guinea-Bissau and the Danish Central Ethical Committee for approval (624-02-0010). The study was registered under clinicaltrials.gov, number NCT00168597.

## Supporting Information

Appendix S1 Trial profile.
(0.03 MB DOC)
