# Supplementary material for: Sex-Differential Effect on Infant Mortality of Oral Polio Vaccine Administered with BCG at Birth in Guinea-Bissau. A Natural Experiment
Source: PLoS One. 2008 Dec 29;3(12):e4056. doi: 10.1371/journal.pone.0004056 (PMC2605256; doi:10.1371/journal.pone.0004056)
Supplement: Appendix S1 — Trial profile. (0.03 MB DOC) [file pone.0004056.s001.doc]

Assessed for eligibility: 4732

Not meeting inclusion criteria for birth weight: 387

Refused to participate: 0

**Enrollment: 4345**

**Allocation**

**Analysis**

**Follow-Up**

**OPV availability**

**Analysis**

Analyzed: 1633

Allocated to vitamin A: 2145

Received vitamin A: 2145

Allocated to placebo: 2200

Received placebo: 2200

OPV at birth:

1663

No OPV at birth:

482

OPV at birth:

1720

No OPV at birth:

480

Died: 14

Never found: 9

Died: 77

Never found: 30

Died: 72

Never found: 27

Died: 16

Never found: 4

Analyzed: 473

Analyzed: 1693

Analyzed: 476
